# Cross-reactivity of eight SARS-CoV-2 variants rationally predicts immunogenicity clustering in sarbecoviruses

**DOI:** 10.1038/s41392-022-01123-7

**Published:** 2022-07-27

**Authors:** Qianqian Li, Li Zhang, Ziteng Liang, Nan Wang, Shuo Liu, Tao Li, Yuanling Yu, Qianqian Cui, Xi Wu, Jianhui Nie, Jiajing Wu, Zhimin Cui, Qiong Lu, Xiangxi Wang, Weijin Huang, Youchun Wang

**Affiliations:** 1 Division of HIV/AIDS and Sex-transmitted Virus Vaccines, Institute for Biological Product Control, National Institutes for Food and Drug Control (NIFDC), WHO Collaborating Center for Standardization and Evaluation of Biologicals, NHC Key Laboratory of Research on Quality and Standardization of Biotech Products and NMPA Key Laboratory for Quality Research and Evaluation of Biological Products, 102629 Beijing, China; 2 Jiangsu Recbio Technology Co., Ltd., 215300 Taizhou, China; 3 Graduate School of Chinese Academy of Medical Sciences & Peking Union Medical College, No. 9 Dongdan Santiao, Dongcheng District, 100730 Beijing, China; 4 CAS Key Laboratory of Infection and Immunity, CAS Center for Excellence in Biomacromolecules, Institute of Biophysics, Chinese Academy of Sciences, 100101 Beijing, China

**Keywords:** Vaccines, Infectious diseases

## Abstract

A steep rise in Omicron reinfection cases suggests that this variant has increased immune evasion ability. To evaluate its antigenicity relationship with other variants, antisera from guinea pigs immunized with spike protein of SARS-CoV-2 variants of concern (VOCs) and variants of interest (VOIs) were cross-tested against pseudotyped variants. The neutralization activity against Omicron was markedly reduced when other VOCs or VOIs were used as immunogens, and Omicron (BA.1)-elicited sera did not efficiently neutralize the other variants. However, a Beta or Omicron booster, when administered as the 4th dose 3-months after the 3rd dose of any of the variants, could elicit broad neutralizing antibodies against all of the current variants including Omicron BA.1. Further analysis with 280 available antigen–antibody structures and quantification of immune escape from 715 reported neutralizing antibodies provide explanations for the observed differential immunogenicity. Three distinct clades predicted using an in silico algorithm for clustering of sarbecoviruses based on immune escape provide key information for rational design of vaccines.

## Introduction

On November 26th, 2021, a new SARS-CoV-2 variant of concern (VOC) was identified by the World Health Organization and named Omicron. The Omicron variant (B.1.1.529, BA.1) was first discovered in Botswana in early November.^1^ Later, a sharp rise in cases of the variant in South Africa’s Gauteng province was reported.^2^ By January 6, 2022, the Omicron variant BA.1 had spread to more than 149 countries, and began rapidly replacing the previously dominant Delta variant all over the world.^3^ There is an increased risk of SARS-CoV-2 reinfection with Omicron, but not Beta or Delta, suggesting that the Omicron variants might more easily evade immunity than any other VOC.^3^

Omicron BA.1 is a highly mutated SARS-CoV-2 variant, containing 35 mutations in Spike protein. In particular, it contains 15 mutations in the receptor binding domain (RBD), including most of the key mutations from previous VOCs and variants of interest (VOIs).^4,5^ It contains the N501Y mutation, which is also found in the Alpha, Beta, and Gamma variants, and is related to increased infectivity, as well as increased mouse ACE2 affinity and immune escape.^6^ It also contains the K417N mutation found in Beta, which was shown to escape neutralization by several monoclonal antibodies.^6^ The E484A mutation of Omicron introduces a different amino acid at a site that is also mutated in Beta, Gamma, and Mu (E484K), which are reported to exhibit significantly decreased neutralization sensitivity to vaccine-elicited sera.^7^ Omicron BA.1 also contains T478K, which is the signature mutation of the Delta variant, and was also shown to facilitate the immune escape of the virus from some monoclonal antibodies.^8,9^ In addition, Omicron BA.1 has important new mutations in key motifs of the RBD, such as N440K, G446S, Q493R, G496S, and Q498R, which may further change the antigenicity of the spike protein.^10^

Preliminary data from multiple studies suggests that Omicron BA.1 is capable of significant escape from the immunity induced by prior infection or vaccination.^11^ However, pressing questions related to the degree of immune escape compared to other VOCs or VOIs, cross-reactivity, as well as the effectiveness of the new generation of vaccines based on Beta or Delta against Omicron, remain unresolved.

In this study, the neutralization range and potency of serum samples collected after immunization with spike proteins from different VOCs and VOIs in guinea pigs were tested and compared using pseudotyped SARS-CoV-2 Omicron, D614G, as well as other VOCs and VOIs. Furthermore, guinea pigs were boosted with Omicron BA.1 or Beta spike protein, and the neutralization before and after the booster dose was also compared. Moreover, we analyzed single or combined mutations in different domains of spike protein to identify the key mutations that determine the antigenicity change of Omicron. Our results provide important clues for scientists to choose immunization strategies against Omicron and possible future variants.

## Results

### Comparing the cross-neutralization activity of antisera against Omicron to other VOCs and VOIs

**Fig. 1:**
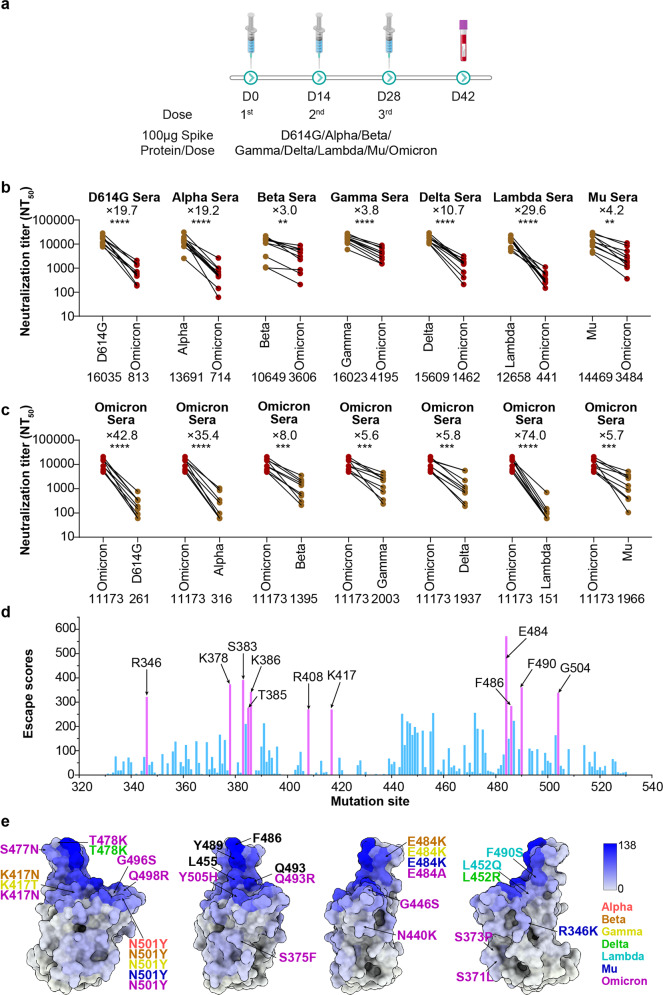
Neutralization activity of sera elicited by Alpha, Beta, Gamma, Delta, Lambda or Mu Spike protein against Omicron pseudovirus. **a** Schematic illustration of the immunization procedure. Guinea pigs were immunized with 100 µg of purified S protein of the D614G, Alpha, Beta, Gamma, Delta, Omicron, Lambda, or Mu variant in three doses. Blood samples were collected 14 days after the third immunization. **b** The neutralization activity of each variant-elicited serum against pseudotyped Omicron was compared with the homologous SARS-CoV-2 variant that was used as the immunogen. Student’s *t* test was used to compare each group with D614G. **c** The neutralization sensitivity of Omicron-elicited sera against pseudotyped Omicron and each pseudotyped variant was compared. Each dot represents the mean NT_50_ of three repeated experiments of one guinea pig serum. The mean NT_50_ values from nine to ten guinea pigs were labeled under the *x* axis. The fold changes are indicated next to the dots. NT_50_, 50% neutralization titer. Student’s *t* test was used to compare each group with Omicron. **d** Site total escape score on SARS-CoV-2 RBD based on 715 reported neutralizing antibodies. The vertical axis represents the total escape score of antibodies at a certain site. The residues with the top 11 highest site escape scores in the histogram are labeled. **e** Surface representation for antigenic heatmap on the RBD. Representative “hot” antigenic residues are labeled. Per residue frequency recognized by the 271 NAbs were calculated and shown. The top eight of the hottest antigenic residues and key residues with substitutions in several VOCs are marked and labeled. Student’s *t* test was used to compare each group with D614G

Guinea pigs were immunized with Spike proteins of D614G, VOCs (Alpha, Beta, Gamma, Delta, and Omicron) or VOIs (Lambda and Mu), and the serum samples were collected 2 weeks after the third immunization. The neutralization activities of the sera were examined using VSV-based pseudoviruses (Fig. 1a). The 50% neutralization titers (NT_50_) of D614G, Alpha, Beta, Gamma, Delta, Lambda, and Mu spike protein-elicited sera against homologous pseudoviruses were 16,035, 13,619, 10,649, 16,023, 15,609, 12,658, 14,469, and 11,173, respectively. The NT_50_ decreased from 16,035 (against D614G) to 813 (against Omicron BA.1) for the D614G-elicited reference serum, representing 19.7-fold reduction. When Alpha or Lambda spike protein was used as immunogen, the neutralization activity for Omicron compared to Alpha and Lambda was decreased 19.2- and 29.6-fold, respectively. The reduction was lower when Delta was used as immunogen, with a 10.7-fold reduced neutralization titer. Interestingly, when Beta, Gamma or Mu was tested, the reduction was even lower, with 3.0-, 3.8-, and 4.2-fold decreased NT_50_ values, respectively (Fig. 1b).

In addition, the neutralizing activity of guinea pig sera elicited by Omicron BA.1 spike protein against pseudotyped Omicron was compared to other variants. Although Omicron BA.1-elicited sera exhibited high neutralization titers against Omicron BA.1 itself (NT_50_ = 11,173), they could not effectively neutralize the other variants. The neutralization activity against D614G, Alpha, and Lambda decreased 42.8-, 35.4-, and 74.0-fold, while the activity against Beta, Gamma, Delta and Mu decreased 8.0-, 5.6-, 5.8-, and 5.7-fold, respectively (Fig. 1c). These results suggested that the immunogenicity of Omicron BA.1 is relatively close to Beta and Gamma, but far from Alpha and Lambda.

RBD, the main target of neutralization in SARS-CoV-2, acts as the most important immunogen during elicitation of neutralizing antibodies. To further unveil the molecular basis of the immunogenic characterizations of these variants, we systematically analyzed the neutralization escape mutation profiles of a total of 715 reported human SARS-CoV-2-neutralizing antibodies using high-throughput yeast-display mutational screening that covers all possible single residue substitutions in the WT RBD background.^4^ Escape scores of the mutations at a particular site on RBD were calculated and used to evaluate the impact of immune evasion on-site mutations for these 715 antibodies (Fig. 1d). The amino acids with the top 11 highest escape scores include residues E484, S383, K378, F490, G504, K386, R346, F486, T385, R408, and K417, among which substitutions of R346, R408, K417, E484, and F486, or combinations of these have been observed in Mu, Beta, Gamma, and Omicron sub-lineages. The total escape scores for the representative variants, defined as the sum of escape scores of all mutations at a particular site on RBD, revealed that Omicron, Beta, Gamma, and Mu confer greater resistance to neutralizing antibodies, which is consistent with in vitro studies using sera for neutralization (Fig. 1b–d). In addition, the antigenic heatmap for RBD using currently available 280 neutralizing antibody complex structures to estimate in vivo antibody-directing frequencies further verified hotspots such as F486, Y489, Q493, L455, E484, and Y505, providing a molecular basis for the differential immune evasion characteristics VOCs and VOIs^4,12^ (Fig. 1e).

### Comparing the cross-neutralization reactivity among different VOCs and VOIs

**Fig. 2:**
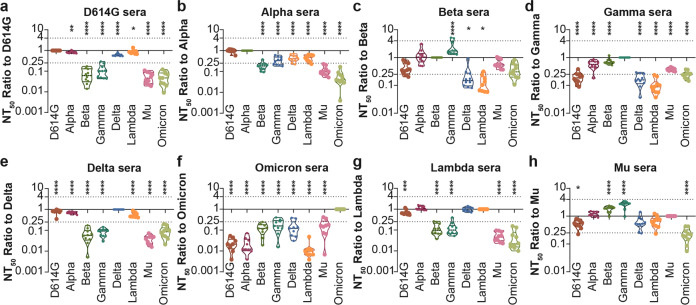
Cross-neutralization analysis of eight Spike protein-elicited sera against eight SARS-CoV-2 variants. The NT_50_ ratios of the tested pseudotyped SARS-CoV-2 variants to the homologous pseudovirus are displayed as a violin plot. The data represent the mean values of three repeated experiments. The dashed line indicates fourfold difference. Data from sera collected after 3 doses of D614G (**a**), Alpha (**b**), Beta (**c**), Gamma (**d**), Delta (**e**), Omicron (**f**), Lambda (**g**), and Mu (**h**). One-way ANOVA and Dunnett’s multiple comparisons tests were used for statistical analysis. The significance of the difference of each group compared to the homogenous immunized group is indicated with asterisks

The relative NT_50_ against each SARS-CoV-2 variant compared to the SARS-CoV-2 variant that was used as the immunogen was calculated to assess the cross-reactivity of the antisera. The results showed that most of the sera (D614G, Alpha, Lambda, Gamma, Delta, and Omicron) showed the best activity against the respective variant that was used as the immunogen. However, the Beta-elicited sera showed slightly higher neutralization activity against Gamma (Fig. 2a–g). Interestingly, Mu-elicited sera showed better neutralization activity against Alpha, Beta, and Gamma than against Mu itself (Fig. 2h). Sera elicited by spike protein of D614G, Delta, and Lambda showed a similar pattern, with almost the same neutralization activity against D614G, Alpha, Delta, and Lambda pseudoviruses but more than tenfold lower activity against Beta, Gamma, Mu, and Omicron pseudoviruses. The neutralization activity of Alpha-elicited sera was similar, with higher activity against Beta and Gamma, which may be due to the presence of the same N501Y mutation. On the other hand, sera elicited by Beta and Gamma spike protein showed the same pattern, with more than tenfold decreased neutralization activity against D614G, Delta, and Lambda pseudoviruses than against the homologous pseudovirus, but less decreased neutralization sensitivity against Omicron and Mu. Surprisingly, Mu-elicited sera displayed equally good protection against almost all the variants (neutralization activity decreased less than fourfold), except for Omicron. These results indicate that the spike protein-elicited sera provide better protection against the pseudotyped SARS-CoV-2 variants with similar mutations as the immunogen. The D614G, Alpha, Delta, and Lambda variants are antigenically similar, while Beta, Gamma, Mu, and Omicron are antigenically closer.

### A booster dose using Beta or Omicron Spike protein as immunogen-induced broad-spectrum neutralizing antibodies

**Fig. 3:**
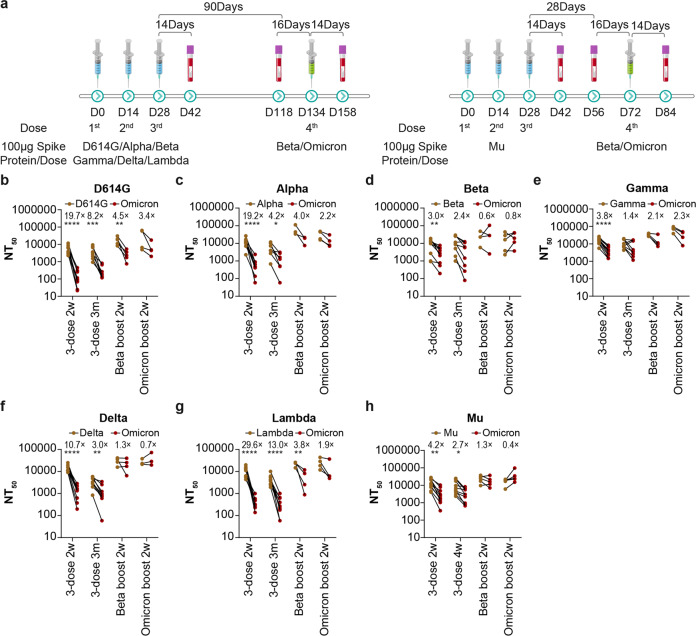
The neutralization antibody titer against Omicron before and after booster administration. **a** Schematic illustration of the booster immunization procedure. Half of the guinea pigs primed with each variant were boosted with a Beta-based immunogen, the other half was boosted with Omicron. **b**–**h** The neutralization activity of each serum against Omicron pseudovirus was compared with the homologous SARS-CoV-2 variant that was used as immunogen. The neutralization activity of sera sampled at 14 days after the third dose, 90 days after the third dose, and 14 days after the fourth dose (Beta/Omicron booster) was compared. Each dot represents the mean NT_50_ of three repeated experiments with one guinea pig serum. The fold changes are indicated next to the dots. Data from sera immunized with 3 doses of D614G (**b**), Alpha (**c**), Beta (**d**), Gamma (**e**), Delta (**f**), Lambda (**g**), and Mu (**h**). Instead of 90 days, guinea pigs were given the booster dose 28 days after the 3rd dose. Student’s *t* test was used to compare the group immunized with the spike protein of the corresponding variant with Omicron

As the antisera elicited by Beta and Gamma spike protein mentioned above provided relatively better protection against Omicron than other variants, several Beta variant-based vaccines are already in development (COVID-19 vaccine tracker and landscape, https://www.who.int/publications/m/item/draft-landscape-of-covid-19-candidate-vaccines) and may be easier to use in an emergency than an Omicron-based vaccine. Accordingly, we next compared the effect of a Beta spike booster to the Omicron spike booster. Half of the previously mentioned guinea pigs which were vaccinated with three doses of spike protein were further boosted with Beta spike protein, while the other half were boosted with Omicron spike protein. Sera were collected 16 days before the 4th immunization (56 days after the 3rd immunization for Mu, and 90 days after the 3rd immunization for other variants), and 14 days after the immunization (Fig. 3a).

**Fig. 4:**
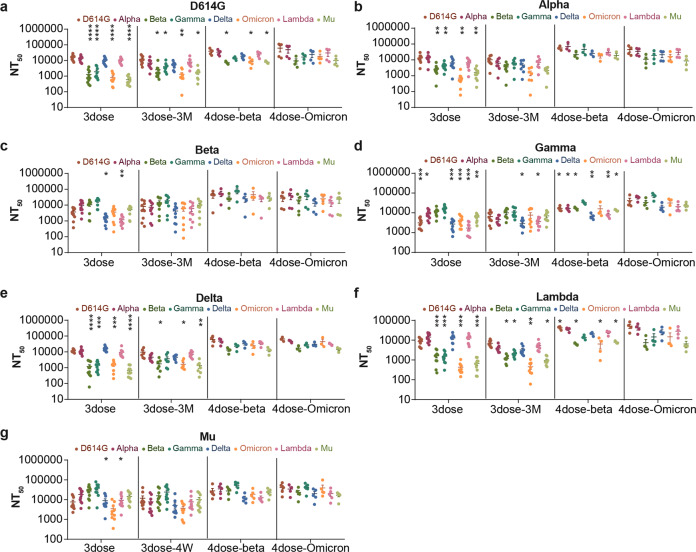
Cross-neutralization analysis of Spike protein-elicited sera before and after Beta/Omicron Spike booster administration. The NT_50_ values of the tested pseudotyped SARS-CoV-2 variants are displayed as a dot plot. The neutralization activity of sera sampled at 14 days after the third dose (three dose), 90 days after the third dose (3dose-3M) and 14 days after the fourth dose (Beta/Omicron booster) were compared (4dose Beta, 4dose Omicron). The data represent the mean values of three repeated experiments. Data from sera elicited by three doses of D614G (**a**), Alpha (**b**), Beta (**c**), Gamma (**d**), Delta (**e**), Lambda (**f**), and Mu (**g**). Two-way ANOVA and Dunnett’s multiple comparisons test were used for statistical analysis. The significance of the difference of each group compared to the group immunized with the spike protein of the corresponding variant is indicated with asterisks

The neutralization activity of sera after either the Beta or Omicron booster increased against not only Omicron (Fig. 3b–h), but also against most of the other tested SARS-CoV-2 variants (Fig. 4a–g).

The ratio of the neutralization activity against Omicron and the variant used as immunogen (first three doses) was also analyzed. The data indicated that the Beta booster led to less severe reduction or a slight increase of the neutralization activity after the booster (0.6–4.5-fold reduction) compared to the sera before the booster (1.4–13.0-fold reduction), while the Omicron booster caused even less reduction or resulted in a slight increase (0.4–3.4-fold reduction, Fig. 3b–h). Interestingly, the neutralization activity of the sera sampled 90 days after the third dose was different from that of sera sampled 14 days after the third dose (Figs. 1b and 4b–h and Supplementary Fig. 1). The neutralization activity against Omicron was less reduced (1.4– 13.0-fold, Fig. 4b–h) at day 90 than day 14 (3.0–29.6-fold, Fig. 1b). This may be due to the obvious decrease of neutralization activity against the homologous variant, combined with the increase of neutralization activity against Omicron at day 90.

The neutralization activity against all the VOCs and VOIs indicated that a booster based on Beta or Omicron could provide broad protection against all the current VOCs and VOIs (Fig. 4b–h).

### The antigenicity relationships of SARS-CoV-2 VOCs and VOIs

**Fig. 5:**
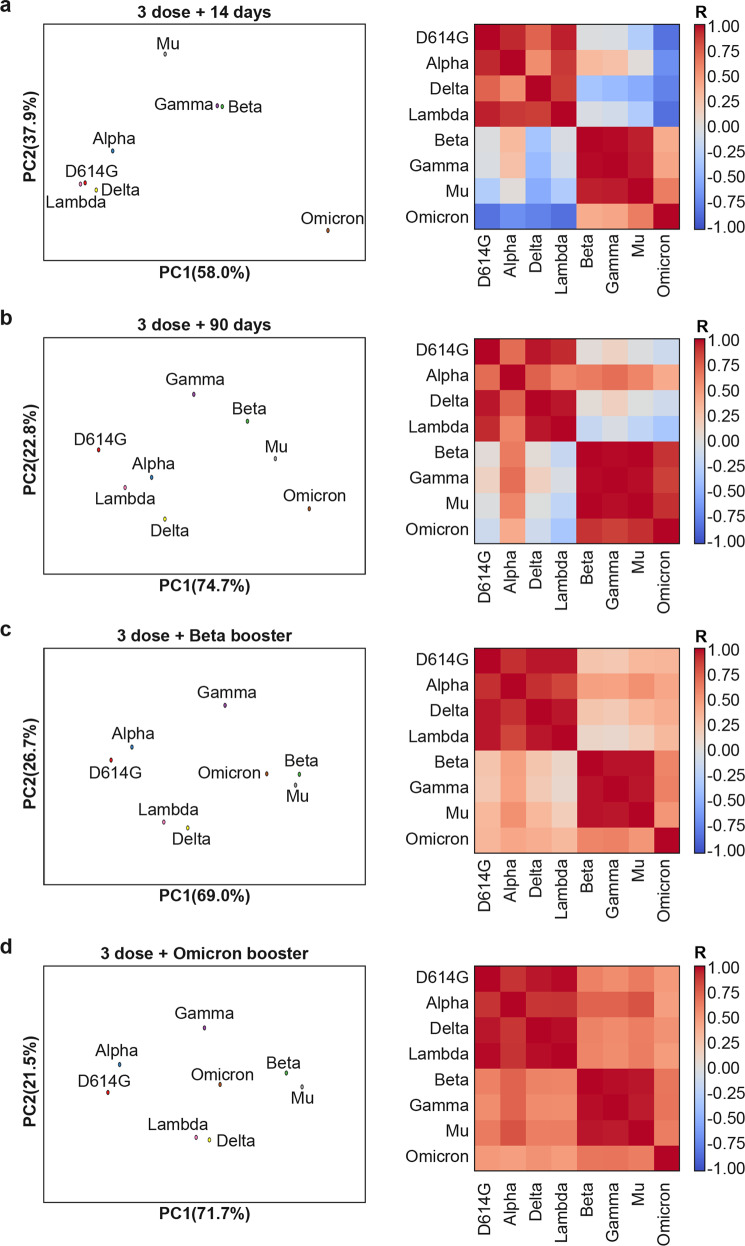
Classification of SARS-CoV-2 spike protein variants by principal component and correlation coefficient analysis. NT_50_ values for each serum/virus pair were log-scale transformed and assembled into vectors for each SARS-CoV-2 variant, resulting in an 8 × 8 matrix. The 1st and 2nd major axes were plotted using Axes3D (left panel). A Spearman correlation coefficient (R) matrix of all virus strains is shown in the form of a heatmap (right panel). The scale bar represents the correlation coefficient. Red indicates a positive correlation between virus variants neutralized by different sera while blue indicates a negative correlation. **a** Neutralization data for sera sampled 14 days after the third dose. **b** Neutralization data of sera sampled 90 days (28 days for Mu) after the third dose. **c** Neutralization data of sera sampled 14 days after the fourth dose (Beta booster). **d** Neutralization data of sera sampled 14 days after the fourth dose (Omicron booster). Instead of 90 days, the booster dose was administered 28 days after the 3rd dose

The antigenicity relationships of the SARS-CoV-2 variants were further analyzed using a method based on antigenic cartography. The antigenic distance was defined by comparing the log NT_50_ for the eight serum/variant pairs. Two principal axes of variation, determined by single-value decomposition of this serum/strain matrix, were displayed to show the distribution of the strains in antigenic space, which provides a simple overview of the antigenic relationships of different variants (Fig. 5a–d, left panel). Spearman correlation coefficients were calculated for each virus strain, and a correlation coefficient matrix is shown in the form of a heatmap (Fig. 5a–d, right panel). These results indicated that the D614G, Alpha, Delta, and Lambda variants are antigenically closer, while Omicron is far from the other variants, but relatively close to Beta (Fig. 5a). The boosters based on Beta, and especially Omicron, shortened the antigenic distance of all variants (Fig. 5c, d).

### The key mutation sites of the Omicron variant that determine its antigenicity

**Fig. 6:**
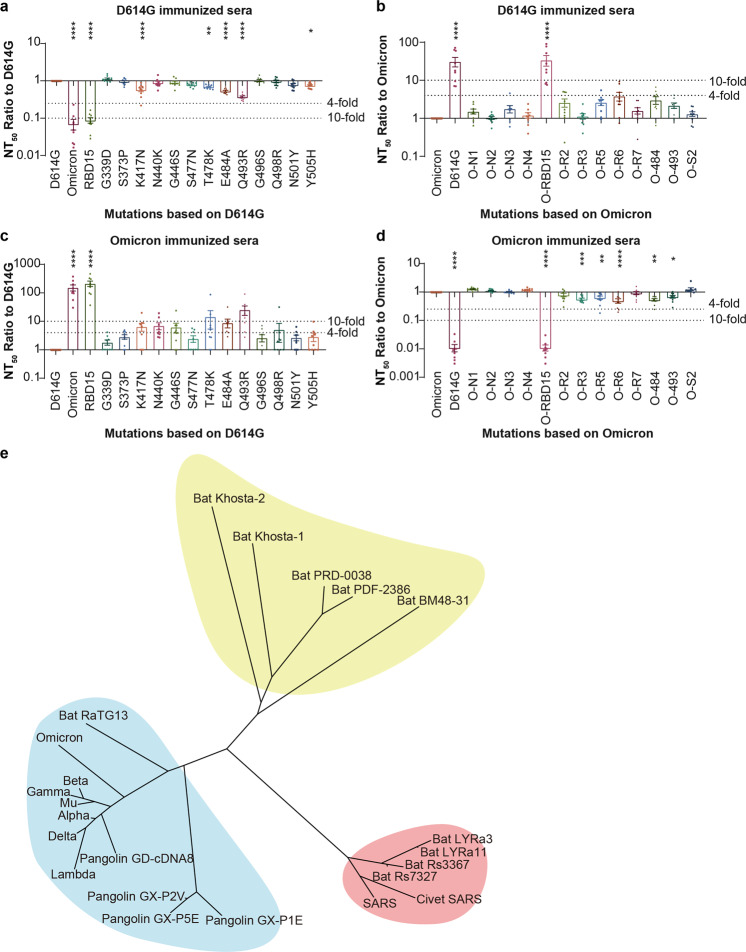
The key mutation sites of the Omicron BA.1 variant that determine its antigenicity and immunogenic clustering of sarbecoviruses. Sera collected from guinea pigs 28 days after three doses of D614G spike immunization (**a/g**) or 14 days after the three doses of Omicron spike immunization (**c/d**) were tested. The column plot shows the NT_50_ titer ratio of each single or combined mutation to D614G (**a/c**) or Omicron (**b/d**). The dashed lines indicate fourfold or tenfold difference. The data represent the mean values of three repeated experiments. **e** Immunogenic clustering of 25 representative sarbecoviruses, including SARS-CoV, SARS-CoV-2 variants and bat- or pangolin-derived coronaviruses based on the site total escape score and amino acid conservation on RBD. One-way ANOVA and Dunnett’s multiple comparisons test were used for statistical analysis

To identify the key mutations that lead to the antigenicity change of Omicron, we compared the neutralization sensitivity of sera elicited by D614G spike protein against RBD15 (D614G with the 15 RBD mutations) and each single mutation in the RBD except for S371L and S375F. The two latter mutations were omitted because the infectivity of the corresponding pseudotyped viruses was too low to be examined, which may due to structural changes resulting in an unstable protein conformation.^13^ The results indicated that the NT_50_ of RBD15 was similar to that of Omicron, and was more than tenfold reduced compared to D614G. The K417N, E484A, or Q493R single mutations resulted in two- to threefold immune escape (Fig. 6a). We next constructed pseudotyped viruses based on Omicron and reversed the mutations to the corresponding amino acids of D614G. The amino acids that determined the antigenicity of SARS-CoV-2 in the RBD domain were divided into six classes in a previous study (Supplementary Table 1).^4^ We then constructed corresponding combined RBD mutations (Supplementary Table 2), and focused on the class I-specific mutations (O_R6, including mutation 417, 496, and 501), class II-specific mutations (O_R7, including mutations 477, 478, 498, and 505), class II and II shared mutations (O_R5, including mutations 484 and 493), class IV specific mutations (O_R2, including mutations 440 and 446), and class VI specific mutations (O_R3, including mutations 339, 371, 373, and 375). The mutations affecting the NTD and S2 domain (including not only mutations located on S2, but also the mutations 547,655,679 and 681, which are located in S1 but not the RBD) were also combined respectively. NTD mutations were separately investigated based on their location (O_N1, mutations 67, 69–70; O_N2, mutation 95; O_N3, mutations 142–145; O_N4, mutations 211–212, 214). The neutralization titers against O-RBD15 (Omicron with the RBD 15 mutations reversed to the corresponding amino acids of D614G) was similar to that against D614G. The neutralization activity against O_R2, O_R5, O_R6, and O_R7 was increased approximately two- to fourfold compared to Omicron, whereas the neutralization activity for O_N1, O_N3, and S2 was also slightly increased (less than twofold, Fig. 6b). The Omicron spike-elicited sera were also tested against the same pseudoviruses (Fig. 3c, d). The neutralizing activity of Omicron-elicited sera against the single mutations of 417, 440, 446, 478, 484, 493, and 498 based on D614G was increased more than fourfold (Fig. 6c), while the activity against O-R3, O-R5, O-R6, O-484, and O-493 based on Omicron was slightly decreased (less than fourfold, Fig. 6d). These results indicated that RBD mutations are the main reason for the immune escape of Omicron, among which 417, 440, 446, 478, 484, 493, 496, and 501 are particularly important. Furthermore, the immune escape was amplified when several important mutations were combined together. The mutations in NTD and S2 may also impact the neutralization sensitivity of Omicron, but only to a very limited degree.

### The antigenic cartography of sarbecoviruses

Having unveiled the molecular determinants of change in antigenicity based on immune escape and analysis of 3D structures in complex with antibodies, we next investigated how this data on alteration in antigenicity and immune evasion could be utilized to develop an in silico algorithm for predicting clustering of sarbecoviruses based on immunogenicity. Given that these key antigenic sites on RBD dominate immune evasion and their immunogenicity, we systematically calculated immunogenic variation scores (*S*_*i,j*_) of pairwise sequences using the equitation: $S_{i,j} = \mathop {\sum}\nolimits_{k = 331}^{529} {B_{ij,k} \times E_{ij,k}}$, where *S*_*i,j*_ is the total variation score of *i* and *j* sarbecovirus variants, the *B*_*ij,k*_ is the similarity score of pairs of residues in the sequence site *k*, and the *E*_*ij,k*_ is the total escape score of the site. After that, we obtained a 25 × 25 dimensional distance matrix and constructed the immunogenic clustering of 25 sarbecoviruses, including SARS, SARS-CoV-2 variants and bat- or pangolin-derived coronaviruses, using the neighbor-joining algorithm^14^ (see “Materials and methods”). As shown in Fig. 6e, the phylogenetic tree could clearly separate these sarbecoviruses into three clades: SARS-CoV-1 related viruses, SARS-CoV-2 variants and more distant bat-derived coronaviruses. Although Omicron is the most striking and a very divergent lineage, moving towards a distinct SARS-CoV-2 serotype, Omicron remains antigenically far distant from the SARS-CoV-1 clade (Fig. 6e). Among classical SARS-CoV-2 lineages, Mu links preceding variants and the recent Omicron, being capable of eliciting more balanced humoral immune responses with increased neutralizing breadth (Fig. 6e). These immunogenic characterizations of sarbecoviruses provide guidance for the rational design of novel broad-spectrum vaccines for protection against sarbecoviruses.

## Discussion

The SARS-CoV-2 virus has continued to evolve since its emergence. Recently, the Omicron variant has been spreading at an unprecedented rate across the world. It has outpaced the Delta variant and become the dominant SARS-CoV-2 strain on the global scale within less than 2 months.^3^ Several recent publications and preliminary data from non-peer-reviewed studies suggest that the Omicron variant has an increased ability of immune escape compared to prior variants.^10,11^ It caused more re-infections than any of the previous variants in those who had recovered from a previous infection or had been vaccinated. There is growing research on vaccine effectiveness against Omicron, which suggests significantly lower neutralization activities against Omicron, including mRNA vaccines,^15^ adenovirus vaccines,^16^ inactivated-virus vaccines, and recombinant protein vaccines.^17^ In addition, most of the previously effective monoclonal antibodies have lost their protective effect against the Omicron variant. A number of pressing questions remain unanswered, including how to respond to the new wave of global pandemic caused by Omicron, how infection or vaccination shapes the immunity against current or future SARS-CoV-2 variants, whether vaccines based on original SARS-CoV-2 strains need to be replaced, and which variant-based vaccine may best fit the current or future needs.

Spike protein-based vaccines such as NVX-CoV2373 from Novavax and ZF2001 form Zifivax were proven to be safe and effective, resulting in their worldwide use.^18^ In this study, we used the spike protein trimer as immunogen, which was expressed by human 293T cells. Six mutations introducing prolines (F817P, A892P, A899P, A942P, K986P, V987P) on S2 and two mutations introducing alanines in the furin cleavage site (R683A and R685A) were introduced to stabilize the protein structure, which was similar to NVX-CoV2373, whereby the 2P mutation (K986P/V987P) on S2 and 3Q mutation at the furin site (682QQAQ-685) were used to stabilize the trimer conformation. Our study will aid the evaluation and development of protein vaccines.

In this study, the cross-reactivity of sera elicited in guinea pigs using spike protein from seven current SARS-CoV-2 VOCs and VOIs as well as the reference strain D614G was analyzed using pseudotyped viruses. Recombinant full-length spike protein was used as immunogen to mimic vaccination or infection. The cross-reactivity was tested not only using sera elicited by other SARS-CoV-2 variants against Omicron pseudoviruses, but also using Omicron BA.1-elicited sera against other pseudotyped SARS-CoV-2 variants. Our data showed that the neutralization titers of sera elicited by the reference strain D614G SARS-CoV-2 decreased significantly against Omicron. This was consistent with previous reports on vaccine-elicited sera, and may be one of the reasons for increased rates of breakthrough infection with Omicron.^10,16^ Furthermore, the neutralization tiers against Omicron of sera elicited by other VOCs or VOIs, especially the Alpha, Delta or Lambda variant, were also obviously reduced, which can also explain the increased incidence of reinfection with Omicron. Moreover, we discovered that Omicron BA.1-elicited sera cannot effectively neutralize the other variants, which indicates that natural infection with only Omicron may not provide useful or broad herd immunity against other SARS-CoV-2 variants. However, when an Omicron-based booster is administered following priming with the initial SARS-CoV-2 vaccine, broadly neutralizing antibodies may be elicited, which may protect against most of the current SARS-CoV-2 variants.

A decrease of neutralizing antibody titer within 3–6 months after vaccination or infection is also suggested to be the reason for recurrent SARS-CoV-2 infection.^19^ Therefore, a booster shot after 6 months was administered in some countries to increase the neutralizing antibody levels and hopefully protect against Omicron. Although a booster dose, especially after a prolonged interval, was proved to greatly increase the neutralizing antibody level,^17,20–22^ repeated booster administration with the same prototype vaccine may not be a good way to fight against Omicron or future new variants, especially for variants with large changes of antigenicity. Our preliminary data indicated that four doses of the D614G vaccine (14-day interval), were not as good as three doses of D614G plus 1 dose of Beta/Omicron (Supplementary Fig. 2). Our immune-cross-reactivity analysis showed that the antigenicity of Omicron is very far from the prototypical D614G strain, but closer to the Beta and Gamma variants. Notably, several vaccine companies have been developing the next generation of vaccines before the Omicron variant appeared, and the antigenicity of Beta is much closer to Omicron. Accordingly, we also tested the idea if a Beta booster can be effective against Omicron. By comparing the Beta booster with an Omicron BA.1-based booster, we found that immunization with Beta spike protein can effectively increase the neutralizing antibodies against Omicron, almost as good as immunization with Omicron spike protein itself.

Interestingly, we also observed that the neutralizing antibody titers against different variants changed at different rates (Supplementary Fig. 1). In the case of D614G-elicited sera, the neutralization activity against D614G, Alpha, Delta, and Lambda pseudoviruses was decreased, while increasing for Beta, Gamma, Omicron and Mu pseudoviruses when comparing sera sampled on day 90 sera to those sampled on day 14 after the 3rd dose. However, the Beta-elicited sera showed a different pattern, since their neutralization activity against most of the variants except Alpha increased at 90 days compared to 14 days after the 3rd dose. The Alpha, Delta, and Lambda-elicited sera were more similar to the D614G sera, while the Gamma sera ere more similar to the Beta sera. Therefore, neutralization activity against the same variant as the immunogen seems to be generated fast, starting at a high level and decreasing at a fast rate. By contrast, neutralizing antibodies against the variant with antigenicity far from the immunogen are elicited slower, at a lower level, but also decreased at a slower rate. This result suggests that there may be a group of antibodies with broader neutralizing activity, which is not easily elicited but may last longer.

Additional Omicron sub-variants emerged after BA.1, including BA.1.1, BA.2, BA.2.13, BA.2.12.1, BA.3, BA.4, and BA.5 (Supplementary Table 3). During the time that this paper was in revision, we tested serum samples (harvested 28 days after the 3rd dose) immunized with the spike of the eight variants against the other Omicron variants that emerged later (Supplementary Fig. 3a–h). Our results indicated that there was no obvious difference when sera elicited by D614G, Alpha, and Lambda were used against BA.1 and BA.2. These results were in accordance with other studies.^23,24^ However, the sera elicited by Omicron BA.1 provided better protection against BA.1 and BA.1.1 than BA.2, BA.2.13, BA.2.12.1, and especially BA.4/5, which is in accordance with the real-world data on re-infections caused by BA.4/5 in individuals who recovered from BA.1 infection, as well as the research showing that Omicron sub-lineages BA.4/BA.5 can escape neutralizing immunity elicited by BA.1 infection.^25–27^ Based on these data, we calculated the distance of the VOCs, VOIs and different Omicron sub-lineages (Supplementary Fig. 3i). Our results indicated that the Omicron variants BA.1, BA.2, BA.2.13, BA.2.12.1, BA.3, BA.4, and BA.5 clustered together. Therefore, the new escape mutants (e.g., R346K, L452R/Q/M) may not greatly change the antigenic distance of Omicron from other variants. Due to the limited availability of sera from day 14, sera from day 28 were used in our later experiments instead. The cluster analysis of D614G and BA.1-elicited sera at days 14 and 28 indicated that there was no difference between the two time points (Supplementary Fig. 3j).

Since SARS-CoV-2 keeps evolving, and Omicron is unlikely to be the last variant, the ultimate goal of vaccines is to elicit immune responses that are broad, strong, and long-lasting, offering protection against epidemic variants and reducing the need for successive booster doses. It is, therefore, crucial to understand the key sites or amino acids that determine the antigenicity of SARS-CoV-2. By using D614G or Omicron (BA.1)-elicited sera, this study analyzed the single or combined mutations of Omicron compared to the reference strain D614G based on the previous epitope analysis. The results suggested that K417N, N440K, G446S, E484A, Q493R, G496S, and N501Y, which are distributed in antigenic classes I, II, III, and IV,^4^ may be important mutations that determine the antigenicity changes of the Omicron variant. Notably, the K417N, E484A/K, and N501Y mutations have been previously identified in the Beta and Gamma variants. This also supported our cross-neutralization results, which suggested that the antigenicity of Beta and Gamma is similar to Omicron. The E484 site is particularly important, as it is shared by antigenic classes I and II, and was repeatedly mutated in Beta, Gamma, Mu, Omicron, and some of the Delta variant isolates. Although the Q493R mutation has never been detected in other VOCs or VOIs, it should be closely monitored in the future, for it is the only mutation that is shared by the antigenic classes I, II, and III, while also leading to a significant change of neutralization sensitivity. Moreover, S371L was reported to affect most of the RBD-directed mAbs.^10,28,29^ However, because the mutation caused structural changes in the spike protein, the pseudoviruses with this single mutation showed a reduced ability to infect the target cells. To compensate for the lack of pseudoviruses with S371L or S375F single amino acid mutations, and the negative effects of single mutations on the whole performance of the virus, we constructed several pseudotyped viruses with combined mutations (Fig. 6) based on recently published structural analyses^4,10^ (Supplementary Table 1). Among them, O_R3 contains the combined reverse mutations of 339-371-373-375. We did not observe obvious changes caused by these mutations. However, our serum study is more broad-based than studies on monoclonal mAbs, and may more closely reflect the actual physiological situation. For example, several single mutations of spike protein may abolish the neutralization ability of several mAbs^7^ (e.g., N501Y, L452R), but have only little influence on sera containing polyclonal antibodies.

The NTD is another important target of neutralizing antibodies, and several studies have reported NTD-specific mAbs (e.g., 4A8, FC05, S2L28) which have broad neutralization activities against different variants.^30–32^ In addition, Liu et al. reported that BA.1 can escape NTD-specific mAbs^28^ (e.g., 4–18, 5–7), which may be related to the mutation of G142D and Del143–145. To examine the role of NTD and S2 in the immune escape of Omicron, the NTD or S2 mutated pseudotyped BA.1 was also reverse-mutated to the prototype (Fig. 6b, d). Only slight effects were seen with D614G- or BA.1-elicited sera, which suggests that immunogenicity may not change much due to NTD or S2 mutations. However, further studies comparing different NTD mutations in different variants using sera from animals immunized with spike protein variants containing NTD mutations may refine and elaborate the role of the NTD.

A major limitation of this study is that only pseudotyped viruses and animal sera were used, and no authentic viruses or human sera from vaccinated individuals were tested. However, pseudotyped viruses have been widely used in previous studies,^10,33^ and the correlation of results obtained using pseudotyped viruses with those obtained using authentic viruses has also been demonstrated.^34^ It is also difficult to obtain a cohort with the same background, immunization doses, and time intervals that covers all the VOCs and VOIs. To accurately compare the cross-reactivity among all the currently important variants, animal experiments seem to be the only option.

Overall, our research indicates that the antigenicity of Omicron is significantly different from the reference strain D614G, and relatively close to the Beta variant. Vaccination using the reference strain D614G as well as pre-infection with Alpha, Delta, or Lambda may not protect against Omicron. Boosters based on Beta or Omicron may offer broad protection against not only Omicron, but also other current VOCs and VOIs. The Omicron-elicited sera also do not protect against other VOCs or VOIs. K417N, N440K, G446S, E484A, Q493R, G496S, and N501Y are the key mutations that changed the antigenicity of Omicron, and should be the focus of studies aiming to develop the next generation of vaccines that elicit broad-spectrum neutralizing antibodies.

## Materials and methods

### Cells

The 293T cells were purchased from the American Type Culture Collection (ATCC, Cat: CRL-3216). The 293T-hACE2 cell line is derived from 293T cells stably expressing human ACE2. The cells were cultured using Dulbecco’s modified Eagle’s medium (DMEM, high glucose; HyClone) supplied with 100 U/mL of penicillin-streptomycin solution (Gibco), 20mM N-2-hydroxyethylpiperazine-N-2-ethane sulfonic acid (HEPES, Gibco) and 10% fetal bovine serum (FBS, Pansera ES, PAN-Biotech), in a 5% CO_2_ environment at 37 °C. The cells were passaged at intervals of 2–3 days using 0.25% Trypsin-EDTA (Gibco).

### Immunization of guinea pigs and serum preparation

Female guinea pigs (9–10 per group, body weight 200–220 g) were immunized subcutaneously with 100 µg of purified S protein of the D614G reference strain, as well as current VOCs (Alpha, Beta, Gamma, Delta, and Omicron) and VOIs (Lambda, Mu) with alum adjuvant once every 14 days for three inoculations. Blood samples were collected 14 days or 28 days after the third immunization.

A booster shot using Beta or Omicron S protein (three to five per group) was administered ~3 months after the third dose (except for the Mu group, in which the interval was 42 days). Blood was collected 16 days before and 14 days after booster administration.

### Ethics statement

The guinea pigs were handled under institutional guidelines for laboratory animal care and use (NIFDC, Beijing, China), and the Animal Care and Use Committee at the NIFDC approved the animal study protocol.

### Construction and production of pseudotyped SARS-CoV-2 variants

The sequence encoding SARS-CoV-2 spike protein was codon-optimized for human cells and cloned into the pcDNA3.1 vector. Site-directed mutagenesis was performed as described previously.^35^ Specific mutation sites and corresponding primers are listed in Supplementary Tables 4 and 5. The pseudotyped viruses were produced by transfecting 293T cells with the S protein expression plasmids and simultaneously infecting them with the G*ΔG-VSV vector (Kerafast, Boston, MA). The supernatant containing the pseudoviruses was harvested at 24 and 48 h post-transfection and stored at −80 for future use.

### Titration of SARS-CoV-2 pseudotyped viruses

The titer of pseudotyped viruses was evaluated by infecting 293T-ACE2 cells with threefold serial dilutions of pseudotyped virus.^36^ The cell culture plate was incubated at 37 °C with 5% CO_2_ for 24 h. Chemiluminescence signals were detected following the protocol of the BriteLite plus reporter gene assay system (Perkin Elmer, Waltham, MA).

### Processing of deep mutational scanning data

The data of deep mutational scanning was obtained from previous research.^10^ The site total escape score of residues, defined as the sum of escape scores of all mutations at a particular site on RBD of each Nabs, was used to evaluate the impact of mutations on each site for all neutralizing antibodies.

### DMS-based Phylogenetic tree

Sequences of RBDs from different variants were aligned with Clustal W.^37^ The immunogenic variation scores (*S*_*i,j*_) of pairwise sequences could be described as the following equitation: $S_{i,j} = \mathop {\sum}\nolimits_{k = 331}^{529} {B_{ij,k} \times E_{ij,k}}$, where *S*_*i,j*_ is the total variation score of *i* and *j* variant, the *B*_*ij,k*_ is the similarity score of pairwise of residues in the sequence site *k*, and the *E*_*ij,k*_ is the site total escape score. The distance of pairwise sequences is defined with Pearson’s correlation, which could be calculated as $d_{i,j} = S_{i,j}^2/S_{i,i} \times S_{j,j}$, where d is the distance score. The distance matrix of variants is used to construct the phylogenetic tree by using Neighbor-joining algorithm from PAUP.^14^

### In vitro neutralization assay with pseudotyped viruses

For the neutralization assays, serial dilutions (starting at 1:30) of serum samples were mixed with 1.3 × 10^4^ TCID_50_ of pseudotyped SARS-CoV-2 variants in 96-well plates at 37 °C for 1 h, then mixed with 293T-ACE2 cells and subsequently incubated for 24 h. The infectivity was determined by measuring the bioluminescence as described above. The 50% neutralization titer (NT_50_) was calculated using the Reed–Muench method.

### Principal component analysis

NT_50_ values for each serum/strain pair were log-scale transformed. Sera from guinea pigs immunized with eight different virus strains were used, assembled on an 8 × 8 matrix. Principal component analysis of this serum/strain pair matrix was carried out, producing eigenvectors representing the axes of variation within the data and each strain. The 1st and 2nd major axes were plotted using Axes3D.^38^ Spearman correlation coefficients were calculated for each virus strain, and a correlation coefficient matrix is shown in the form of heatmap.

### Statistical analysis

GraphPad Prism 8 (GraphPad, San Diego, CA) was used for statistical analysis. Values are shown as means ± standard error of the mean (SEM). Unpaired two-tailed Student’s *t* test was used for comparisons of two groups of data. One- or two-way ANOVA and Dunnett’s multiple comparisons test were used for statistical analysis of multiple groups of data. Significance thresholds were as follows: *P < 0.05, **P < 0.01, ***P < 0.005, and ****P < 0.001.

## Supplementary information

supplymentary material

## Data Availability

Original data for Figs. 1–6 have been deposited to figshare: https://figshare.com/projects/Cross-reactivity_of_eight_SARS-CoV-2_variants_rationally_predicts_immunogenicity_clustering_in_sarbecoviruses/142697.
