# Supplementary material for: Cross-reactivity of eight SARS-CoV-2 variants rationally predicts immunogenicity clustering in sarbecoviruses
Source: Signal Transduct Target Ther. 2022 Jul 27;7:256. doi: 10.1038/s41392-022-01123-7 (PMC9328627; doi:10.1038/s41392-022-01123-7)

Supplementary Materials for

Cross-reactivity of eight SARS-CoV-2 variants rationally predicts immunogenicity clustering in sarbecoviruses

Qianqian Li, Li Zhang, Ziteng Liang, Nan Wang, Shuo Liu, Tao Li, Yuanling Yu, Qianqian Cui, Xi Wu, Jianhui Nie, Jiajing Wu, Zhimin Cui, Qiong Lu, Xiangxi Wang, Weijin Huang and Youchun Wang

.

Correspondence to: xiangxi@ibp.ac.cn, huangweijin@nifdc.org.cn, wangyc@nifdc.org.cn

**This PDF file includes:**

Figures. S1 to S3

Tables. S1 to S5


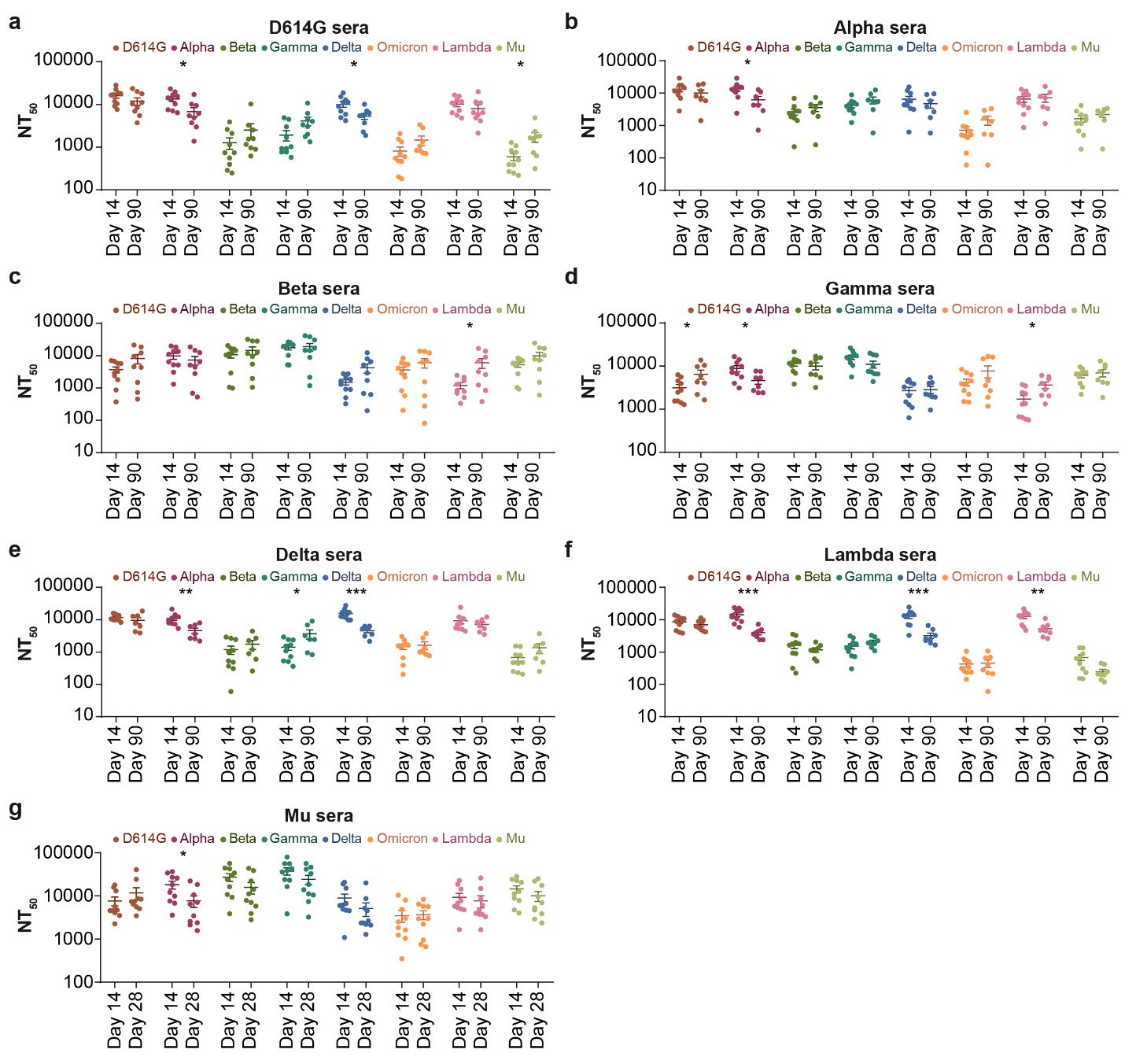


Figure. S1. The Kinetics of NT_50。_

**(a-g)** Dot plot showing the NT_50_ values of sera sampled 14 or 90 days after the 3^rd^ dose (except in (**g)**, were serum sampled after 28 instead of 90 days was used for Mu). Each group of immunized sera was analyzed against each of the eight SARS-CoV-2 pseudoviruses as indicated. Data are derived from three repeated experiments. Student’s *t*-test was used for statistical analysis. The significance of the difference between the Day 90/28 and Day14 groups was indicated in each column.


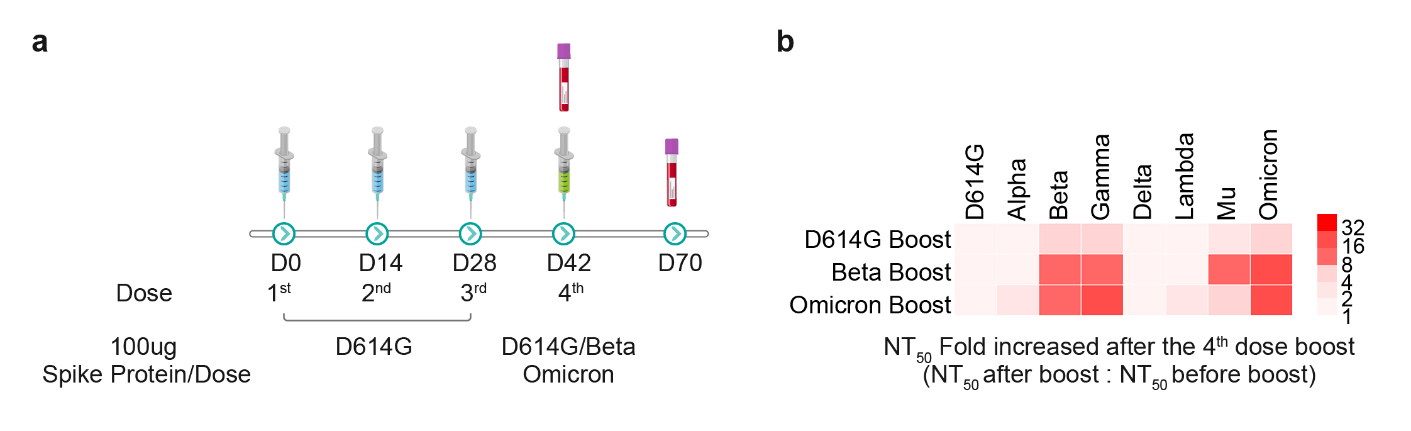


**Figure. S2. Comparison of D614G, Beta, and Omicron Boosters.**

**(a)** Schematic illustration of the booster immunization procedure. Twelve guinea pigs primed with 3 doses of D614G spike protein, were divided into three groups (four per group), and boosted with D614G, Beta or Omicron spike protein. Serum samples were collected before booster injection (14 days after the 3^rd^ dose) and 28 days after the booster dose.

**(b)** The neutralization activity of each serum sample against eight pseudotyped viruses was examined. NT_50_ of the serum harvested before the 4^th^ dose booster and after the 4^th^ dose booster against the same pseudotyped virus was compared. The fold increase after the 4^th^ dose booster was displayed as a heatmap, with darker red color indicating a higher fold increase.


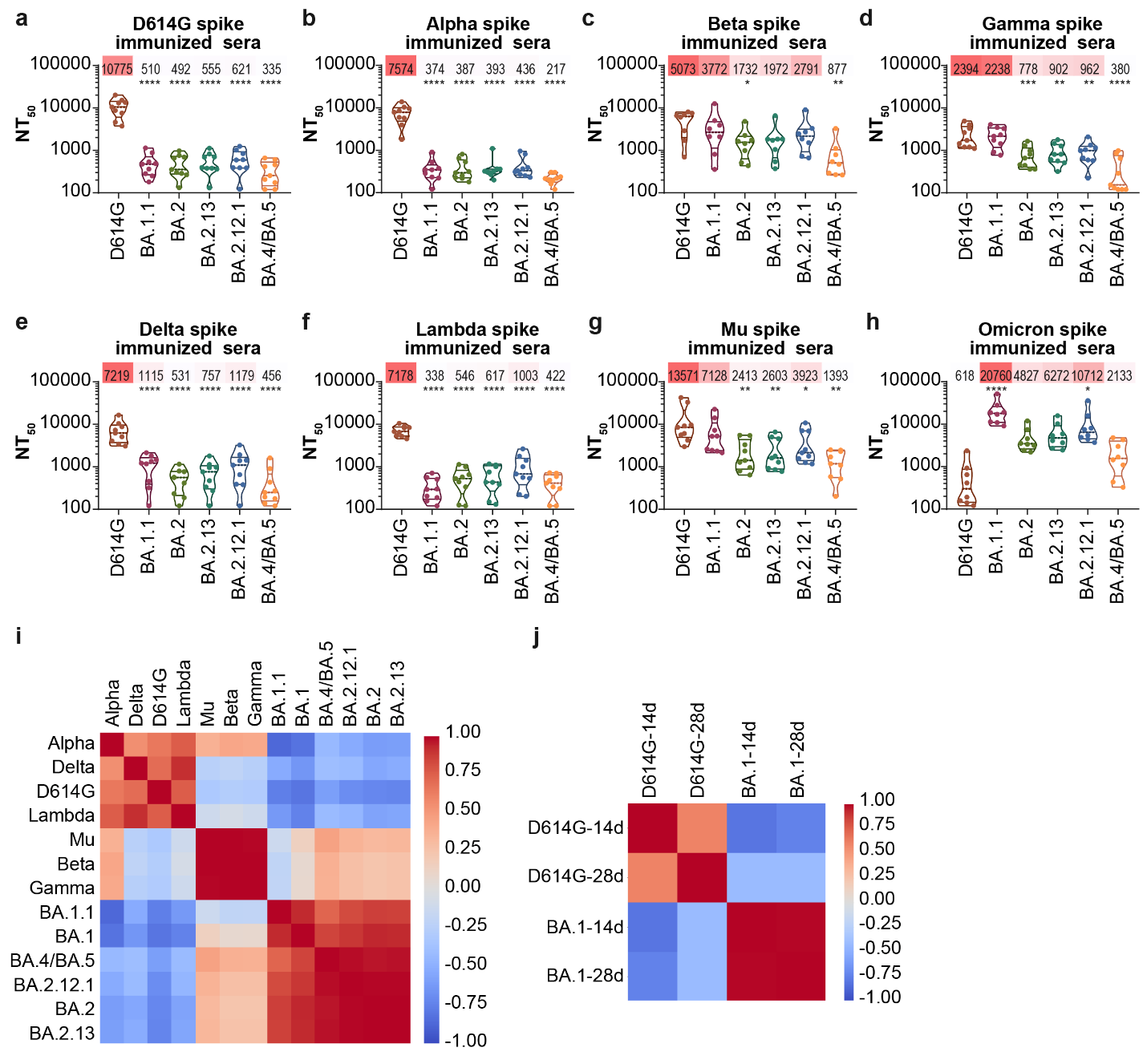


Figure. S3. The immunogenicity of Omicron sub-lineages.

**(a-h)** The neutralization activity of each variant-elicited serum (harvested 28 days after the 3^rd^ dose) against pseudotyped Omicron sub-lineage variants was examined. The data represent the mean NT_50_ values of three repeated experiments. Data from sera elicited by D614G (**a**), Alpha (**b**), Beta (**c**), Gamma (**d**), Delta (**e**), Lambda (**f**), Mu (**g**) and Omicron (**h**). The mean NT_50_ values were indicated in the column. **i-j.** Correlation coefficient analysis of virus antigenicity.

A Spearman correlation coefficient (R) matrix of all virus strains is shown in the form of a heatmap. The scale bar represents the correlation coefficient. Red indicates a positive correlation between virus variants neutralized by different sera while blue indicates a negative correlation.

Table S1. Antigenic classes of the mutation sites on SARS-CoV-2 spike protein.


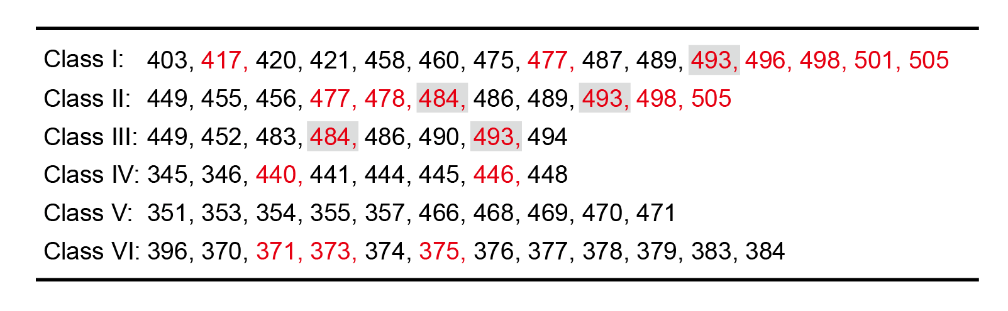


Table S2. Pseudotyped viruses with combined mutations based on Omicron.


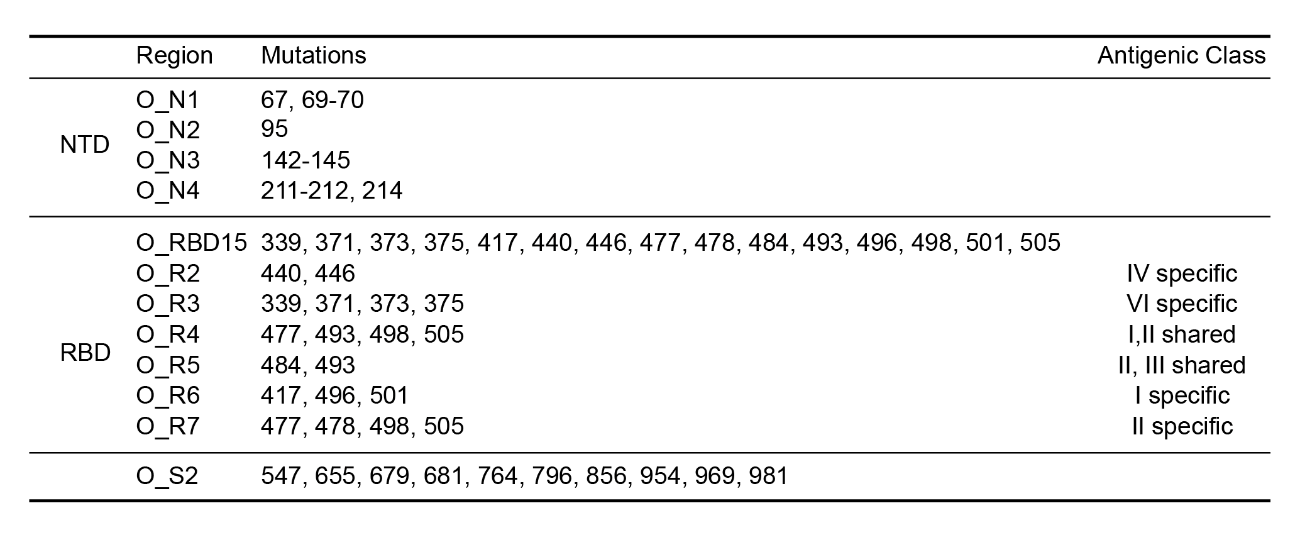


Table S3. Mutations on the Spike protein of Omicron sub-lineages.


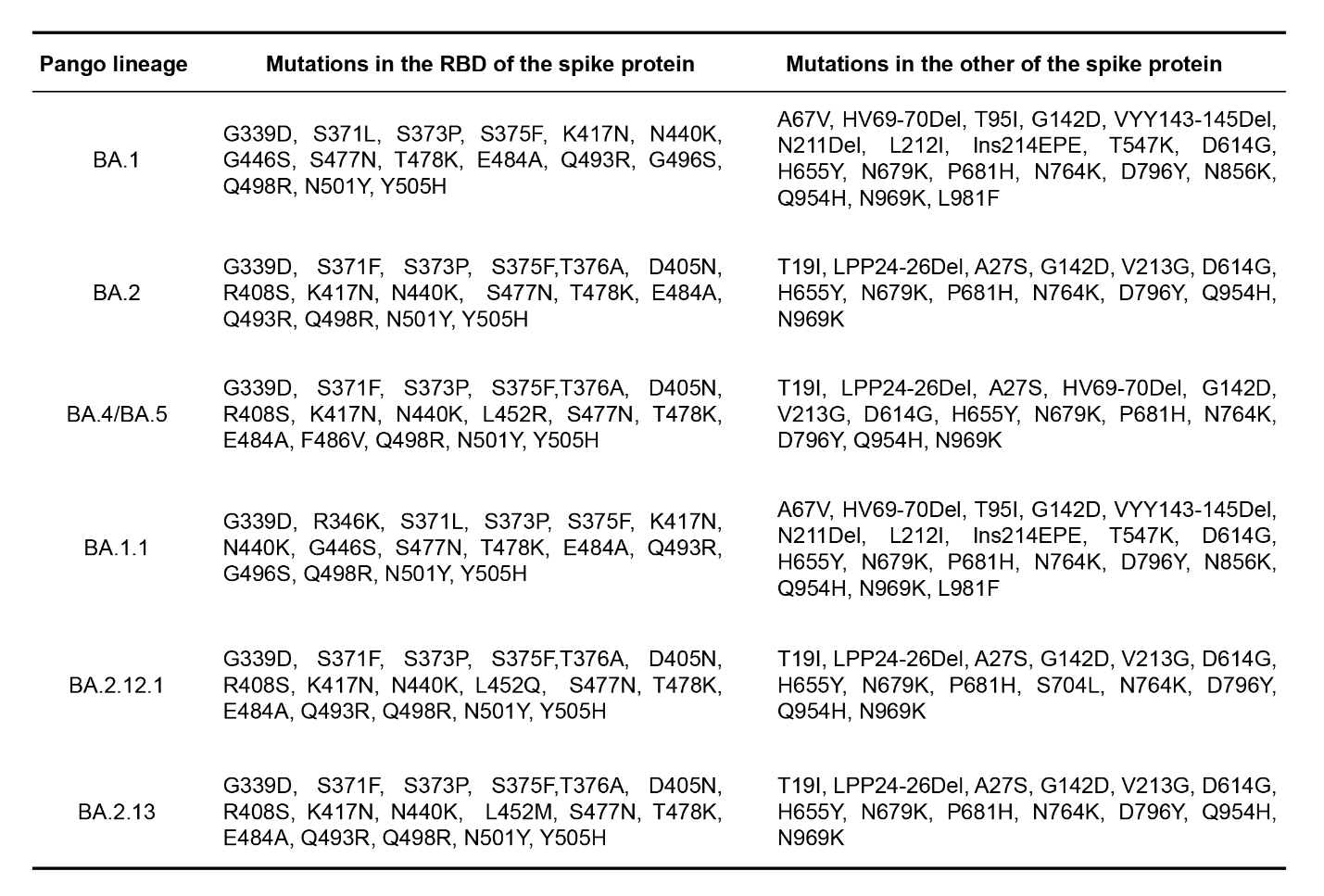


Table S4. Mutations on the Spike protein of VOCs and VOIs.


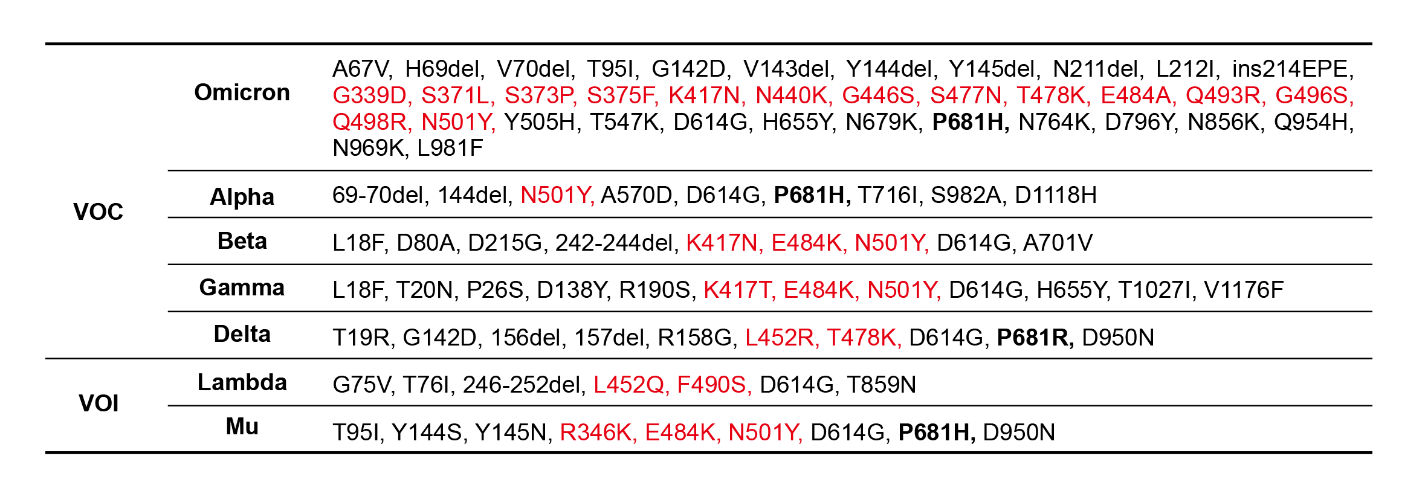


Table S5. Primers used for site-directed mutagenesis.


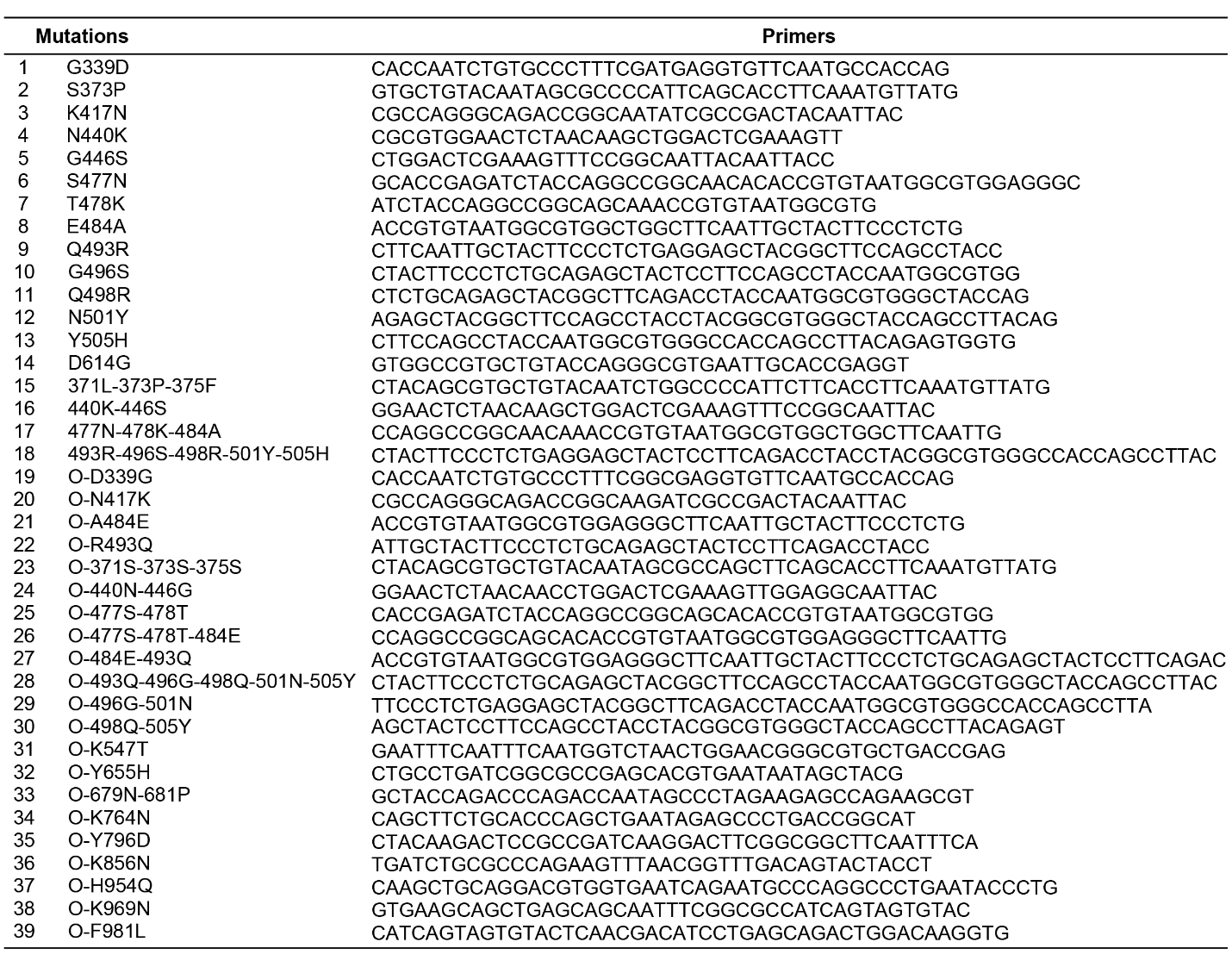

Supplement: Supplementary file 1 — supplymentary material [file 41392_2022_1123_MOESM1_ESM.docx]
